# Post translational modifications of Trifolitoxin: a blue fluorescent peptide antibiotic

**DOI:** 10.1038/s41429-021-00497-0

**Published:** 2022-01-12

**Authors:** Benjamin J. Lethbridge, Robert E. Asenstorfer, Laura S. Bailey, Brenda T. Breil, Jodie V. Johnson, Graham P. Jones, Victor Rumjanek, James J. Sims, Max E. Tate, Eric W. Triplett

**Affiliations:** 1 School of Agriculture, Food and Wine, University of Adelaide, Waite Campus, Urrbrae, SA 5064 Australia; 2 Department of Chemistry, University of Florida, P.O. Box 117200, Gainesville, FL 32611-7200 USA; 3 PK Yonge Developmental Research School, University of Florida, Gainesville, FL 32601-7845 USA; 4 Mass Spectrometry Research and Education Center, Chemistry Department, University of Florida, Florida, FL 32611-7200 USA; 5 Departamento de Química, Universidade Federal Rural do Rio de Janeiro, Seropédica, 23851-970 RJ Brazil; 6 Department of Plant Pathology, University of California, Riverside, CA 92521 USA; 7 Department of Agronomy, University of Wisconsin-Madison, Madison, WI 53706 USA; 8 Department of Microbiology and Cell Science, University of Florida, Gainesville, FL 32611-7200 USA

**Keywords:** Small molecules, Peptides

## Abstract

Trifolitoxin (TFX, C_41_H_63_N_15_O_15_S) is a selective, ribosomally-synthesized, post-translationally modified, peptide antibiotic, produced by *Rhizobium leguminosarum* bv. *trifolii* T24. TFX specifically inhibits α-proteobacteria, including the plant symbiont *Rhizobium* spp., the plant pathogen *Agrobacterium* spp. and the animal pathogen *Brucella abortus*. TFX-producing strains prevent legume root nodulation by TFX-sensitive rhizobia. TFX has been isolated as a pair of geometric isomers, TFX1 and TFX2, which are derived from the biologically inactive primary amino acid sequence: Asp-Ile-Gly-Gly-Ser-Arg-Gln-Gly-Cys-Val-Ala. Gly-Cys is present as a thiazoline ring and the Arg-Gln-Gly sequence is extensively modified to a UV absorbing, blue fluorescent chromophore. The chromophore consists of a conjugated, 5-membered heterocyclic ring and side chain of modified glutamine.

## Introduction

More than half a century ago, a low molecular weight, papain-sensitive antibiotic that confers a competitive advantage for its producing strain *Rhizobium leguminosarum* bv*. trifolii* T24 in the nodulation of clover was reported [1]. It was subsequently named Trifolitoxin (TFX) by Triplett and Barta [2]. Constitutive production of TFX has been described by Triplett and Barta [2] and Triplett [3]. Its significance stems from the fact that it inhibits most strains of a specific group of α-protobacteria including nitrogen fixing symbiotic bacteria in the genus *Rhizobium*, plant pathogens from *Agrobacterium* spp. as well as the animal pathogen *Brucella abortus* [4]. TFX production can be transferred to a wide range of α-proteobacteria by incorporation of the tfxABCDEFG cassette of genes [4]. TFX production in *Rhizobium etli* improves this strain’s ability to occupy bean root nodules under agricultural conditions [5]. Trifolitoxin is known as a linear azol(in)e containing peptide (LAP) [6–9] but a complete structure remains to be elucidated.

The eleven C-terminal amino acid sequence (DIGGSRQGCVA) of the 42 amino acid residue structural gene product of TfxA [10] is consistent with the reported degradative amino acid sequence of purified, blue fluorescent, trifolitoxin (DIGGSRXGCVA) [6] post-translational modification of the predicted 42mer prepeptide. Synthesis and testing of the unmodified non-fluorescent undecapeptide failed to show any toxicity to TFX-sensitive strains. Conservative substitution of amino acids RQGC by site-directed mutagenesis eliminated toxicity [11] also indicating further post-translational modification or involvement of these amino acids in the activity or synthesis of TFX.

Here we report the structure of TFX2 and spectroscopic differences to TFX1 isolated as a pair of geometrical isomers, TFX1 and TFX2, which elute in that order from a reverse phase HPLC column. TFX2 is much more active than TFX1 [11] and the two isomers are interconvertible. Post-translational modifications of amino acids and adjacent peptide backbones of the eleven C-terminal amino acids of the pre-peptide produces a blue fluorescent trifolitoxin.

## Methods

### Bacterial strains and strain culture

*R. leguminosarum* bv. viceae 128C1, ANU794 [11] or 309 [12] was used as the sensitive strain in TFX bioassays according to the method of Schwinghamer and Belkengren [1] or Scupham and Triplett [11]. Strains T24 and 128C1 were cultured on Bergersen’s synthetic medium [13] with 1.5% noble agar (Difco Laboratories, Detroit, Michigan, USA). This medium is referred to as BSM-N.

### TFX purification and bioassay

TFX1 and TFX2 were purified as described previously [14].

### High voltage paper electrophoresis and paper chromatography

High voltage paper electrophoresis and paper chromatography were done according to Tate [15] and Smith [16].

### Ultraviolet spectroscopy

Ultraviolet spectra were recorded on a Perkin Elmer Lambda 5 spectrophotometer. For pKa determination, UV spectra of TFX2 were recorded over the range pH 1-13.

### Amino acid composition and sequencing

Sequencing was obtained by the automated Edman method or by manual Edman degradation according to the method of Klemm [17] and the subtractive method of Konisberg [18]. The amino acid composition of the fragment peptides was determined by complete acid hydrolysis, high voltage paper electrophoresis at pH 1.7 and ninhydrin staining. Thiols were determined with Ellman’s reagent [19]. Thiol oxidation was done using performic acid [20]. Hydrazinolysis was determined by the method of Frankael-Conrat and Tsung [21].

### Enzymatic digestion of TFX

Proteolytic fragments of TFX were generated by incubating the sample preparation of Sigma insolubilised protease in 50 mM phosphate pH 7.0 for 24 h at 28 °C. An amount of TFX corresponding to 0.2 A_302_ was added to 1 mg protease. The insoluble protease was removed by centrifugation and fragments isolated by high voltage paper electrophoresis. All other proteolytic digestions were prepared as described by Smyth [22]. Arginase and amino acid hydrolysates from TFX were incubated in 50 mM Na_2_HPO_4_ (pH 9.7) from 0 to 40 min [23].

### Mass Spectrometry

High resolution mass spectra (HR-MS) of TFX1 & TFX2 were obtained by Fast-atom bombardment mass spectrometry (FAB-MS) using a ZAB-ET tandem mass spectrometer (VG Analytical Ltd, Manchester, UK). The samples were embedded in a matrix containing 3-benzyl alcohol, glycerol and 1% trifluroacetic acid. HR- MS results were confirmed by Bruker Impact II UHR-Qq-TOF-MS (Ultra-high resolution Quadrapole-quadrapole Time-of-flight mass spectrometer; Bruker Daltonik GMBH, Bremen, Germany) controlled by Hystar Software 3.2 SR4 (version 3.2; Bruker Daltonik GMBH) operated in in positive electrospray injection ((+)-ESI) mode. Separation of TFX1 and TFX2 was carried out with a Ultimate 3000 series HPLC (Thermo Scientific, Waltham, Massachusetts, U.S.) constituting of degasser, binary pump, column compartment and autosampler controlled by Dionex Chromeleon Xpress software (Thermo Scientific). Chromatographic separation was achieved on a ThermoScientific Acclaim PepMap RSLC (300 µm × 15 cm, 2 µm, 100 Å) with a flow rate of 5 microliters/min at 40 ^o^C column temperature. Mobile phases A and B were water and methanol, respectively, each with 10 mM ammonium formate and 0.1% formic acid. The following gradient was used; 2% B (0–2 min) increased to 10% B at 21 min, increased to 95% B at 40 min and held at 95% B for 10 min.

Initial tandem mass spectrometry (MS/MS) data were attained by FAB-MS. Further, MS/MS data were obtained by reversed phase gradient C18 HPLC/(+) ESI-MS (electrospray ionisation mass spectrometry) and (+)ESI-MSn (electrospray tandem mass spectrometry). Samples were dissolved in water. (a) Mass spectra were obtained using a ThermoFinnigan (San Jose, CA, USA) LCQ in electrospray ionisation (ESI) mode. HPLC separation was achieved using an Agilent (Palo Alto, CA, USA) 1100 series binary pump and a Waters (Milford, MA, USA) Symmetry Shield RP18 (2.1 x 150 mm) column. (b) (+)ESI-MS/MS of *m/z* 519 [M + 2H]^2+^ ions obtained using a ThermoScientific LTQ XL linear quadrupole ion trap mass spectrometer with electrospray ionisation (ESI) operating with XCALIBUR 2.2 SP1.48. Separation of TFX-1 and TFX-2 by Thermo Scientific Ultimate 3000 HPLC (Waltham, MA, USA) with dual pumps, column heater and auto-sampler using a Thermo-Scientific HyperSil Gold aQ (2.1 × 150 mm; 3 µm) column at 35 ^o^C. Mobile phases were (A) 10 mM ammonium acetate and 0.1% formic acid in water and (B) 10 mM ammonium acetate and 0.1% formic in water:methanol (1:9). (c) Finally high resolution MS/MS was performed on the Bruker Impact II UHR-Qq-TOF-MS. ACD/Chemsketch [24], ProteinProspector [25], ChemBioDraw [26] and Molecular Weight Calculator [27] were used to aid in interpretation of the MS/MS spectra.

### Nuclear magnetic resonance spectroscopy

NMR data for TFX1 and TFX2 were collected on a Bruker Avance 500 MHz spectrometer at the National Magnetic Resonance Facility at Madison (NMRFAM). Samples were purified as described above and resuspended in either 100% D_2_O or 90% H_2_O/10% D_2_O, and TSP (sodium 3-trimethylsilylpropionate) was added as a ^1^H chemical shift reference (0.00 ppm). All NMR data were collected at 5 °C to minimise sample degradation. For samples dissolved in 90% H_2_O, water signal was reduced by presaturation. The following two dimensional (2D) data were collected: TOCSY [28], ROESY [29], ^13^C-HSQC [30], and ^13^C-HMBC [31]. The TOCSY mixing sequence was DIPSI-2 [32] using a 7.1 kHz field strength and a mixing time of 32 ms. The ROESY mixing time was 300 ms with a continuous wave field strength of 2.8 kHz. Data were processed using FELIX [33] and analysed using NMRView [34] and MestReNova [35]. Resonance assignments in the unmodified regions of TFX1 and TFX2 were done with standard ^1^H-based methods [36].

### Molecular modeling

Models of TFX1 and TFX2 were constructed using BioChemDraw [26] and energy minimised by MM2 within Chemdraw3D [37]. To aid NMR interpretation, ^1^H and ^13^C were predicted using MestReNova [35] software.

## Results

### Primary amino acid structure of TFX

**Fig. 1:**
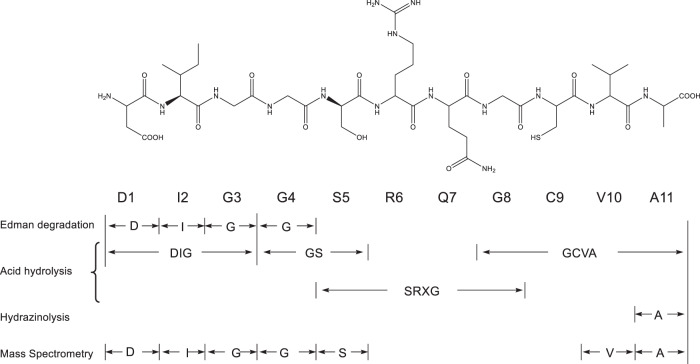
Unmodified TFX peptide with the results of the chemical analyses of the biologically active TFX peptide

Amino acid analysis (as PTH derivatives) of complete hydrolysates of TFX (1 and 2) showed the presence of three Gly residues and one residue each of Asp, Ile, Ser, Arg, Val, Ala, and Cys (as half cystine) (Fig. 1). TFX did not react with the thiol reagents *N*-ethylmaleimide or Ellman’s reagent which indicated the cysteine thiol group was not free. Presence of a thiazoline ring was suggested by (a) the thiol group could only be detected with mild acid hydrolysis, (b) the UV chromophore observed in strong acid (10 N HCl) was similar to that of the protonated thiazoline in bacitracin, glutathione and 2-methyl thiazoline and (c) the ionisation constant (pKa ≈ −1) for nitrogen protonation was similar to bacitracin thiazoline (pKa −1.8; ref. [38]).

Acidification of TFX (0.1 N HCl/1 h) followed by performic acid oxidation converted the cysteine of TFX to cysteic acid, TFXSO_3_H, which retains fluorescence and partial biological activity. Digestion of TFXSO_3_H with an insolubilised non-specific protease liberated Asp, Ile, Gly, Val, Ala, Ser, and yielded several blue fluorescent fragments, which could readily be detected on paper electrophoretograms with a UV lamp (254 nm). Acid hydrolysis of the smallest protease-derived, fluorescent-fragment yielded Arg, Gly, cysteic acid, and an unidentified ninhydrin-positive component by high voltage paper electrophoresis (HVPE) consistent with an acidic amino acid, but with more negative charge than glutamic acid. The amino acid sequence obtained by Breil et al. [10], suggested glutamine was part of the fluorescent chromophore which however after acid hydrolysis would be observed as glutamic acid. The HVPE result indicated that glutamine has undergone further modification. This established that arginine and glycine and the modified glutamine are involved in chromophore formation. It is reasonable to suggest, that prior to post-translational modification all the identified amino acids of TFX, apart from glycine have the l configuration. Papain and carboxypeptidase digestion of TFX eliminated TFX antibiosis, but aminopeptidase and trypsin had little or no effect. It should be noted that aminopeptidases have a very low specificity for N-terminal aspartic acid [23]. The inability of trypsin to eliminate biological activity, originally noted by Schwinghamer and Belkengren [1], is uncommon for a peptide containing arginine and indicated an unusual peptide bond following that residue. Arginine was also isolated as a d-l racemic mixture (resolved by arginase treatment) indicating the potential for an adjacent conjugated system. Automated Edman degradation of TFX produced the N-terminal sequence Asp-Ile-Gly-Gly before the reaction sequence falters. Hydrazinolysis showed that the C-terminal amino acid was alanine. Partial acid hydrolysis (5 N HCl, 5 min, 110 ^o^C) gave identifiable peptides of DIG, GS, SRXG and GCVA. Together these data are consistent with the amino acid sequence, DIGGSRXGCVA.

High resolution fast atom bombardment mass spectrometry (FAB-MS) yielded *m/z* values of 1038.4392 and 1038.4403 Da for the MH^+^ species of TFX1 and TFX2, respectively. These isomers are interconvertible (40 ^o^C, 44 h) and both fit the formula C_41_H_64_N_15_O_15_S suggesting a geometric rearrangement. These MS results were confirmed by ultra-high resolution (+)-ESI MS which gave *m/z* 519.72402 ± 0.00064 and *m/z* 519.72395 ± 0.00079 for MH^2+^ ions or neutral mass of 1037.44804 and 1037.44790 for TFX1 and TFX2, respectively (Table S2). High-resolution and low-resolution tandem mass spectrometry of both peptides indicated the first five residues on the N-terminus were unmodified with the sequence Asp-Ile-Gly-Gly-Ser and the C-terminal peptides were unmodified Val-Ala (Supplementary S7-S26). This in agreement with the findings of Liu [39]. There was no evidence from the mass spectrometry for an unusual linkage between the serine and arginine that was indicated by the Edman degradation. The peptide RXGC does not give ions predicted for an unmodified peptide. A single observation by FAB-MS of an ion at 725 *m/z* suggested that cysteine in the terminal peptide CVA has lost H_2_O.

TFX1 and TFX2 produced *m/z* 1038.4 [M + H]^+^ ions, but an unmodified peptide with the sequence DIGGSRQGCVA would have produced an *m/z* 1062.5 [M + H]^+^ ion. Thus, post-translational modifications to convert DIGGSRQCVA into TFX cause a decrease of 24 mass units which is equivalent to one dehydration (−18) and three oxidations (-6H).

The proton NMR for TFX 1 indicated there was one proton in the alkene/olefinic region (*δ*_H_ 5.69 ppm) and 46 protons in the aliphatic region which consisted of ten methine protons (*δ*_H_ 1.83–4.76 ppm), one methine proton shifted upfield by a sulphur atom to *δ*_H_ 5.21 ppm, twenty methylene protons and fifteen methyl protons. COSY proton NMR allowed the identification of seven protons in the amide region (*δ*_H_ 8.2–8.8 ppm). Twenty-five carbons peaks could be identified by ^13^C-HMBC and ^13^C-HSQC spectra which included five methyl carbons (*δ*_C_ 13.120 ppm) and ten methylene (*δ*_C_ 27.0–63.7 ppm) and eight methine carbons (*δ*_C_ 33.0–69.9 ppm). Two carbons were in the region associated with alkynes (*δ*_C_ 75,100 ppm). A carbon at *δ*_C_ 80.1 ppm was consistent with a cysteine α-carbon. The remaining unsaturated carbon (*δ*_C_ 83.5 ppm) was associated with the proton in the alkene/olefinic region (*δ*_H_ 5.69 ppm). The NMR data for proton and carbon spectra of TFX-2 were virtually identical to TFX-1 except for the proton in the alkene region which shifted from *δ*_H_ 5.69 ppm to *δ*_H_ 5.80 ppm and an amide proton shifting from *δ*_H_ 8.12 ppm to *δ*_H_ 7.99 ppm (Fig. S26–S27).

NMR analyses were consistent with mass spectroscopic results and the chemical modifications described above. From the pattern of chemical shifts, it is clear that TFX2 and TFX1 are similar molecules (Table 1 and 2). Using standard ^1^H-directed sequential assignment methods [36] the amino acids aspartic acid (D1) through arginine (R6) were unambiguously assigned in both TFX molecules. However, the sequential assignments for both molecules stop with arginine α-proton (R6-H^α^). The arginine α-proton was rapidly exchanged when the NMR data were collected in 100% D_2_O, showing that it is unusually acidic and can be expected to be easily racemised above pH 8 where biological activity is also lost. Additionally, analysis of arginine obtained from acid hydrolysates of TFX-sulfonic acid indicated equal proportions of d- and l-arginine.

**Table 1:** ^1^H and ^13^C NMR, COSY and HMBC correlations for TFX2

Amino acid	Carbon	*δ*^13^C	Proton	*δ*^1^H	COSY	HMBC
Asp-1			HN	nd		
	Cα	53.3	Hα	4.26–4.22 (m, 1H)	Hβ1, Hβ2	
	Cβ	40.4	Hβ1	2.80 (dd, *J* = 17.0, 5.6 Hz, 1H)	Hα	Cα, COOH
			Hβ2	2.69 (dd, *J* = 17.0, 7.8 Hz, 1H)	Hα	
Ile-2			HN	8.70	Hα	
	Cα	61.6	Hα	4.20 (d, *J* = 7.0 Hz, 1H)	HN, Hβ	Cβ, Cδ, Cγ, C=O
	Cβ	38.7	Hβ	1.92-1.84 (m, 1H)	Hα, Hγ1, Hγ2	Cδ
	Cg	27.3	Hγ1	1.51–1.44 (m, 1H)	Hβ, Hδ, Hγ2	
			Hγ2	1.20 (dt, *J* = 13.4, 7.9 Hz, 1H)	Hβ, Hγ1	
	Cδ	17.3	Hδ	0.93 (d, *J* = 6.8 Hz, 3H)	Hβ, Hγ1 Hε	Cγ, Cα
	Cε	13.1	Hε	0.87 (t, *J* = 7.4 Hz, 3H)	Hβ, Hγ1, Hγ2	Cγ, Cβ
Gly-3			HN	8.70	Hα	
	Cα	45.2	Hα	3.97 (d, *J* = 2.7 Hz, 2H)		
Gly-4			HN	8.37	Hα	
	Cα	45.0	Hα	4.03 (d, *J* = 6.7 Hz, 2H)		
Ser-5			HN	8.41	Hα	
	Cα	58.4	Hα	4.51 (t, *J* = 5.7 Hz, 1H)	HN,Hβ	C=O
	Cβ	63.7	Hβ	3.89–3.83 (m, 2H)	Hα	
Arg-6			NOH	8.53		
	Cα	58.0	Hα	4.76–4.72 (m, 1H)	Hβ	
	Cβ	33.1	Hβ1	1.98–1.91 (m, 1H)	Hα, Hγ1	
			Hβ2	1.92–1.84 (m, 1H)	Hγ1, Hγ2	
	Cγ	27.0	Hγ1	1.72–1.64 (m, 1H)	Hβ1, Hβ2, Hδ	Cα
			Hγ2	1.64–1.57 (m, 1H)	Hβ1	
	Cδ	43.2	Hδ1	3.19 (t, *J* = 6.8 Hz, 2H)	Hγ1	C(NH)NH2
Chr			NH	nd		
	Cγ	83.7	Hγ	5.80 (s, 1H)		Cα, Cβ, Cδ
Gly-8			HN	nd		
	Cα	43.6	Hα1	4.46 (d, *J* = 17.6 Hz, 1H)		
			Hα2	4.61 (d, *J* = 17.4 Hz, 1H)		
Cys-9			HN	nd		
	Cα	80.1	Hα	5.19 (t, *J* = 9.3 Hz, 1H)	Hβ1, Hβ2	
	Cβ	38.2	Hβ1	3.70 (dd, *J* = 11.3, 9.7 Hz, 1H)	Hα	Cα, C=O
			Hb2	3.53 (dd, *J* = 11.4, 8.4 Hz, 1H)	Hα	
Val-10			HN	7.99		Hα
	Cα	61.7	Hα	4.17 (d, *J* = 6.8 Hz, 1H)	HN, Hβ	Cγ, Cβ, C=O
	Cβ	33.0	Hβ	2.08 (h, *J* = 6.7 Hz, 1H)	Hα, Hγ1	Cα, Cβ, Cγ2
	Cγ1	21.2	Hγ1	0.89 (d, *J* = 6.8 Hz, 3H)	Hβ, Hγ2	Cα, Cβ, Cγ1
	Cγ2	19.8	Hγ2	0.79 (d, *J* = 6.8 Hz, 3H)	Hγ1	
Ala-11			HN	8.2		Hα, Hβ
	Cα	53.9	Hα	4.10 (q, *J* = 7.3 Hz, 1H)	HN, Hβ	Cβ, COOH
	Cβ	20.0	Hβ	1.32 (d, *J* = 7.2 Hz, 3H),	Hα	Cα

Carbons derived from HSQC and HMBC, amide protons from COSY

*Chr* chromophore

**Table 2:** ^1^H and ^13^C NMR, COSY and HMBC correlation for TFX1

Amino acid	Carbon	*δ*^13^C	Proton	*δ*^1^H	COSY	HMBC
Asp-1			HN	nd	Hβ1, Hβ2	
	Cα	53.2	Hα	4.30–4.25 (m, 1H)	Hα	COOH
	Cβ	40.3	Hβ1	2.81 (dd, *J* = 17.1, 5.6 Hz, 1H)	Hα	Cα
			Hβ2	2.71 (dd, *J* = 17.0, 7.7 Hz, 1H)		
Ile-2			HN	8.69		
	Cα	61.7	Hα	4.20 (d, *J* = 7.0 Hz, 1H)	Hβ1, Hδ	C=O
	Cβ	38.7	Hβ	1.91-1.83 (m, 1H)	Hα, Hγ1,Hγ2, Hδ, Hε	C=O
	Cg	27.4	Hγ1	1.52–1.43 (m, 1H)	Hα, Hγ2, Hδ, Hε	
			Hγ2	1.19 (dt, *J* = 14.1, 7.9 Hz, 1H)	Hα, Hγ1, Hδ, Hε	
	Cδ	17.4	Hδ	0.92 (d, *J* = 6.8 Hz, 3H)	Hβ, Hγ1, Hγ2	Cα, Cβ Cγ
	Cε	13.1	Hε	0.86 (t, *J* = 7.4 Hz, 3H)	Hβ, Hγ1, Hγ2	Cδ
Gly-3			HN	8.71		
	Cα	45.2	Hα	3.97 (d, *J* = 6.6 Hz, 2H)	Hα	
Gly-4			HN	8.39	HN	
	Cα	45.1	Hα	4.03 (d, *J* = 5.0 Hz, 2H)		
Ser-5			HN	8.44	Hα	
	Cα	58.4	Hα	4.51 (t, *J* = 5.6 Hz, 1H)	HN, Hβ	C=O
	Cβ	63.7	Hβ	3.87 (d, *J* = 6.2 Hz, 2H)	HN, Hβ	
Arg-6			NOH	8.52		
	Cα	57.9	Hα	4.78 (dd, *J* = 8.5, 5.9 Hz, 1H)	Hβ1, Hβ2, Hγ1, Hγ2,	
	Cβ	33.1	Hβ1	1.99–1.92 (m, 1H)	Hα, Hγ1, Hγ2	
			Hβ2	1.91-1.83 (m, 1H)	Hα, Hγ1, Hγ2	
	Cγ	27.0	Hγ1	1.71–1.65 (m, 1H)	Hα, Hβ1	Cα
			Hγ2	1.65–1.56 (m, 1H)	Hα, Hβ2,	
	Cδ	43.1	Hδ1	3.19 (t, *J* = 7.0 Hz, 2H)	Hβ1, Hβ2, Hγ1, Hγ2	
Chr			NH	nd		
	Cγ	83.5	Hγ	5.69 (s, 1H)		Cβ, Cδ
Gly-8			HN	nd		
	Cα	43.5	Hα1	4.46 (d, *J* = 17.2 Hz, 1H)		
			Hα2	4.63 (d, *J* = 17.2 Hz, 1H)		
Cys-9			HN	nd	Hβ1,Hβ2	
	Cα	80.1	Hα	5.21 (t, *J* = 9.0 Hz, 1H)	HN, Hβ2	
	Cβ	38.2	Hβ1	3.70 (t, *J* = 10.7 Hz, 1H)	HN	Cα, C=O
			Hb2	3.55 (dd, *J* = 11.4, 8.4 Hz, 1H)		
Val-10			HN	8.12	HN, Hβ	
	Cα	61.9	Hα	4.17 (d, *J* = 7.0 Hz, 1H)	Hα, Hβ	Cβ, C=O
	Cβ	33.0	Hβ	2.08 (dt, *J* = 13.6, 6.7 Hz, 1H)	HN, Hγ1, Hγ2	
	Cγ1	21.1	Hγ1	0.89 (d, *J* = 6.9 Hz, 3H)	Hβ	Cβ
	Cγ2	19.9	Hγ2	0.81 (d, *J* = 6.8 Hz, 3H)	Hβ	
Ala-11			HN	8.20	Hα, Hβ	
	Cα	53.8	Hα	4.11 (q, *J* = 7.2 Hz, 1H)	HN, Hβ	COOH
	Cβ	20.0	Hβ	1.33 (d, *J* = 7.2 Hz, 3H),	HN, Hα	Cα, COOH

Carbons derived from HSQC and HMBC, amide protons from COSY

*Chr* chromophore

Absence of an arginine amide NH in the COSY spectrum (Fig. S24) and the arginine α-carbon is shifted downfield 58.0 ppm compared with the value predicted by MestReNova [35], 52.1 ppm, suggested the arginine amide was substituted with oxygen (Table 1). The observed putative arginine N–(O)–H proton (8.53 ppm) corresponds to that expected for a secondary hydroxamic acid [40]. In the TFX2 TOCSY data (Fig. S32) a downfield shift of the NOH proton to 8.95 ppm is consistent with trans to cis isomerisation of the hydroxamic acid [41]. Further evidence for presence of a hydroxamic acid is the racemisation of the arginine alpha-proton. The results from the Edman degradation of TFX also suggest that the bond between serine and arginine was not behaving like a normal amide. Pseudobase formation can occur in many nitrogen-containing heterocycles [42] with an approximate hydration constant ($pK_R^ +$) of 2.1 estimated using the methods of Perrin et al. [43], and Bunting [42]. Rapid dehydration of the hydroxyl and the arginine α-proton during ionisation of mass spectrometry gives the observed mass of *m/z* 1038.4.

Even though the peptide and *tfxA* DNA sequence analyses showed that TFX contains a cysteine residue, freshly isolated TFX did not react with Ellman’s reagent for free –SH groups, indicating the presence of a bound thiol group. However, incubation of TFX at low pH (0.1 M HCl) caused a characteristic rise in absorbance at 410 nm on reaction with Ellman’s reagent, as a result of a free SH group and a decrease in a UV band characteristic for thiazoline rings near 270 nm in 10 N HCl. This reaction has been utilised for the convenient calculation of the molarity of TFX in solution. Below pH 5.0 biological activity declined rapidly to about two percent or less of the original activity as the thiazoline ring opened. According to Schmir [44] Δ^2^-thiazolines hydrate at low pH to form a pseudobase which is in equilibrium with the ring open species and therefore it was possible to estimate hydration constant for the TFX thiazoline ring by spectroscopic method, pK_H_ 2.61 ± 0.05. Four lines of evidence support the notion that the cysteine in TFX is cyclized to form a thiazoline ring. First, a free thiol group is detected only after mild acid hydrolysis. Second, the ultraviolet spectrum of TFX in 10 M HCl is very similar to that of the thiazoline ring in bacitracin. Third, TOCSY NMR data from TFX2 clearly show two species in solution, one lacking an alpha-amide proton for the cysteine and a second with an amide-proton (Fig. S32). This strongly suggests that a mixture of peptides with an intact thiazoline ring and the ring-opened normal cysteine residue were present during NMR analyses. Fourthly, the FAB-MS of TFXSO_3_H had a molecular mass, *m/z* 1104.3, which is 18 Da (H_2_O) greater than would be expected (*m/z* 1086.4) for simple oxidation.

The TOCSY and ROESY spectra for TFX2 cis-hydroxamic acid isomer show two distinct sets of similar resonances, identified in Fig. S32 as V10*, V10, A11*, and A11. Since two of the similar resonances were clearly alanine, correlations were examined beginning with each of these peaks and sequentially stepped towards the N-terminus. The spin system labelled “A11” was preceded by a valine, which is labelled “V10” (Fig. S32). The amide proton of V10, in turn, is correlated to a proton, labelled C9 (Hα). The proton C9 (Hα) is correlated to a pair of Hβ protons at 3.7 and 3.6 ppm, but the identity of an amide proton correlated with C9 (Hα) could not be determined in the TFX2 TOCSY spectrum. Thus, the sequential assignments for the C9-V10-A11 group stopped on the N-terminal end with Hα of C9. This pattern of chemical shifts is consistent with a thiazoline ring.

The ultraviolet spectrum of the isomeric mixture TFX (1 and 2) and the purified isomers TFX1 and TFX2 exhibited characteristic peaks of 239 and 302 nm at pH 7. Molar extinction coefficients, determined by the SH concentration after acid hydrolysis of the thiazoline ring structure using Ellman’s reagent were ε 7,200 and ε 13,800 at 302 nm and 239 nm, respectively. The shorter wavelength maximum shifts to 228 nm upon acidification with a pKa of 4.95 (±0.04) (Fig. S6). Dehydration of the hydroxamic acid moiety would extend the bond conjugation to produce a chromophore consistent with a blue fluorescence (254 nm excitation) observed on the HVPE paper after drying (Figs. S2, S3).

DNA sequence analysis, mass spectrometry, and NMR analyses described above indicate that this chromophore is derived from glutamine. The fluorescence of this chromophore suggests that the glutamine has modified during post-translational modification. NMR sequential analysis from both the N- and C-termini accounted for all residues, except glutamine, the amide proton of G8, and the amide proton of C9 in the thiazoline-containing species. The assignments from the N-terminus ended at Arg Hα, which was also found to be acidic. The only other observable proton in the NMR spectrum of TFX2 not accounted for by NMR sequential analysis was a vinyl proton resonating at 5.80 ppm, which is unusual for peptides and was directly bonded to a carbon resonating at 82.4 ppm (Table 1). This vinyl proton has no neighbouring protons within four covalent bonds, as evidenced by an uncoupled peak in the proton 1D spectrum and by a lack of correlations in the TOCSY spectrum. In addition to the directly bonded carbon at 82.35 ppm, we observed three robust ^13^C HMBC correlations to the vinyl 5.80 ppm proton: 114.7 ppm, 168.6 ppm, 177.0 ppm. These NMR data are consistent with the enolic form of 3-keto glutamine with an unsaturated α-carbon. No glutamine amide proton was observed in the COSY spectrum which is consistent with this data.

Absence of an amide proton for glycine (G8) in either TOCSY or COSY spectra suggest this amide nitrogen participates in the formation of the chromophore. An absorbance maximum of 302 nm is indicative of conjugation of four double bonds [45]. The (*Z*)-3-hydroxybut-2-enamide portion of the 3-keto glutamine contributes two double bonds with the unsaturated glutamine α-carbon adding a third. To obtain further conjugation, either the glycine amide nitrogen or the glutamine amide nitrogen must be unsaturated. To form a stable chromophore, cyclisation between the glycine amide nitrogen and the arginine amide carbonyl similar to the cyclisation involved in the formation of green fluorescent protein [46] would give a chromophore with the desired characteristics. An ionisation constant (pKa) of 4.95 (±0.04) was consistent with the phenol on the resulting imidazole ring [43].

In the ROESY spectra, the TFX2 chromophore proton (5.80 ppm) is correlated to a proton at 4.5 ppm. This could either be the two glycine (G8) protons at 4.61 ppm and 4.46 ppm or the serine α-proton at 4.50 ppm (Fig. S38). The proton at 4.50 ppm is also correlated with protons at 3.81 ppm and a proton at 8.72 ppm/8.36 ppm. Areas calculated from the ROESY data indicate that there are two protons at 3.81 ppm and one proton at 8.72 ppm/8.36 ppm and one at 5.90 ppm which is consistent with the chromophore proton being correlated with the serine proton. Furthermore, molecular modelling (MM2; ref. [38]) indicates the distance between the chromophore proton and the serine α-proton is 3.81 Å which is in the range for a NOE correlation (Fig. S40). This can only occur when the chromophore proton is in the trans-configuration. TFX1 shows no ROESY through-space correlation with the serine proton and an upfield shift of the chromophore proton to 5.62 ppm is consistent with a cis-configuration (Fig. S41). Further evidence for interaction of the chromophore with the serine is a change in the NMR coupling of the serine β-protons from a multiplet (TFX2) to a doublet (TFX1) indicating a change from restricted to free rotation of the α–β carbon bond.

Besides the chromophore proton, only the valine amide protons show a significant difference in shift between TFX1 (8.12 ppm) and TFX2 (7.99 ppm). This results from large structural changes in the peptide secondary structure due to the cis-trans isomerisation in the chromophore. According to our current modelling [38] the distance between the valine amide carbonyl and the hydroxyl proton on the imidazole ring for TFX2 is 6.74 Å but only 3.79 Å for TFX1 which would be close enough for weak hydrogen bonding with a concomitant increase in the C–N double bond character consistent with the downfield shift observed.

## Discussion

**Fig. 2:**
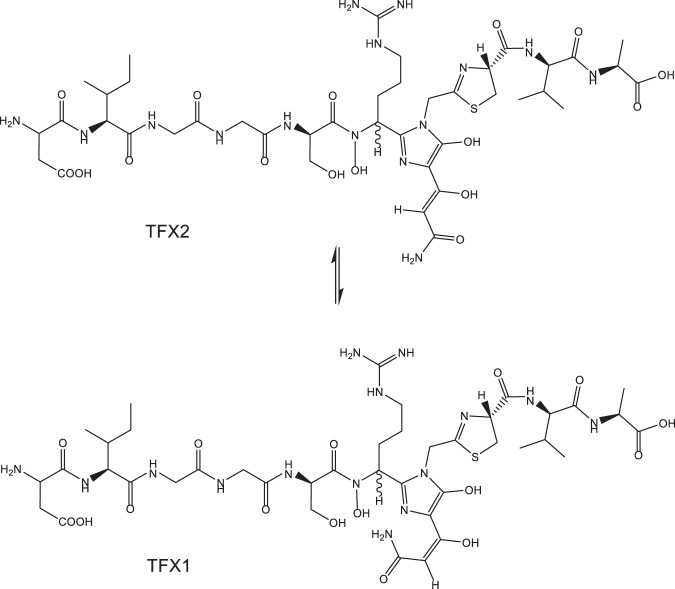
Structure of TFX in aqueous solution

TFX is a highly unusual undeca-peptide that has been post-translationally modified in three positions (Fig. 2). That together with multiple isomeric forms has made structural elucidation difficult. Trifolitoxin is one of the most highly characterised peptide antibiotics produced by any α-proteobacterium. This along with phazolicin, a linear azol(in)e-containing peptide (LAP) from *Rhizobium* sp. Pop5 (ref. [9]) makes post-translationally modified peptides an interesting source of antibacterials. Each of the four modified amino acids (RQGC) in the TFX peptide is required for TFX activity. TFX1 (cis-isomer) of TFX has much lower biological activity than TFX2 (trans-isomer) [11]. Scupham and Triplett [11] made numerous amino acid substitutions in the peptide by site-directed mutagenesis and found that any substitution in the RQGC fragment, including conservative changes, resulted in loss of TFX activity. These authors also concluded that both the thiazoline and the UV-absorbing chromophore, are important for antibiotic activity. The unique structure of the chromophore may play a role in its narrow specificity, but the mode of action is unknown.

The glutamine sidechain in TFX has been modified to the enolate tautomer of 3-oxo-glutamine, an oxidation product of 3-hydroxy-glutamine. 3-Hydroxy-glutamine is also a part of the structure of the antifungal pneumocandin B0 from *Glarea lozoyensis* [47] and is a derivative of the biologically significant amino acid, 3-hydroxy glutamic acid. This amino acid belongs to the family of β-substituted amino acids of fungal origin which include tricholomic acid (*Tricholoma* species) [48] and its oxidation product ibotenic acid (*Amanita muscaria*) [49, 50]. Reduction and hydrolysis of ibotenic acid gives an acidic amino acid profile on ninhydrin stained electrophoretograms very similar to reduced and hydrolysed fluorescent peptide fragments of TFX. Ibotenic acid has a dissociation constant (pKa of 5.1) similar to the currently reported pKa of the TFX chromophore, but lack of other supportive evidence for this molecular rearrangement of the glutamine side chain, including an absence of decarboxy ibotenic acid (muscimol) in acid hydrolysates of TFX has ruled out an ibotenyl derivative. Clearly reduced hydrolysed products of the glutamine derivative of TFX and ibotenic acid are closely related and so 3-oxo-glutamine is a very suitable candidate as part of the chromophore.

Other known glutamine derivatives such as pyroglutamic acid [8] can be ruled out as these only occur as an N-terminal amino acid and glutamine in TFX is present as an intra-peptide amino acid. Peptide hormones in mammalian systems, such as the thyrotropin-releasing hormone, are known to contain modified N-terminal glutamine residues that are present as pyroglutamic acid [51–53] however, no intra-peptide modifications of glutamine similar to the TFX chromophore have been reported. The modification of the glutamine residue in TFX is novel and appears to be essential for its narrow bacteriocin-like spectrum of activity.

A series of known enzymatic or spontaneous chemical reactions could convert the glutamine residue to the blue fluorescent chromophore (Fig. S42). In the case of TFX, a pathway of oxidation (dehydrogenation) followed by cyclization is favoured, because each of the steps necessary for oxidation are known and well characterised, and the resulting structure would be energetically well-poised for formation of the GFP-like chromophore. Our first proposed step is enzymatic oxidation of the glutamine Cα-Cβ bond to β-keto glutamine. The β-keto glutamine creates geometry analogous to a GFP-like ring closure in which the nitrogen of glycine acts as a nucleophile in the cyclization with the carbonyl carbon of arginine. Following proton transfer, water would be lost in the formation of a new double bond between the arginine carbonyl carbon and glutamine nitrogen followed by spontaneous oxidation to complete the GFP-like portion of the chromophore [54]. Subsequent rearrangement, where there is a shift of the double bond from between the arginine α-carbon and carbonyl carbon to between the arginine α-carbon and amide nitrogen. In aqueous solution, this double bond is hydrated to give the hydroxamic acid.

Thiazoline (or its thiazole oxidation product) structures are quite well known in peptides of bacterial origin, bacitracin (*Bacillus licheniformis*) and thiostrepton (*Streptomyces* sp.) being good examples. Bacitracin is a non-ribosomally synthesized antibiotic [55], thiostrepton [56, 57] and phazolicin [9] are ribosomally synthesized and post-translationally modified peptides like trifolitoxin. The thiazoline of TFX is very acid sensitive and this feature alone has been very useful in identifying this type of backbone modification in TFX. In TFX, a cyclodehyratase catalyses the thiazoline ring closure [39]. The thiazoline is required for maximal biological activity. Conversion of the thiazoline to the thiol, disulphide and the sulfonic acid reduced activity in the bioassay to 1-2%, 0.05-0.1% and 1-2%, respectively of original activity [6].

The chemical properties of this peptide now explain some important biological observations. For example, unlike the vast majority of known antibiotics produced by bacteria, trifolitoxin is produced constitutively rather than only in stationary phase. Given the instability of the thiazoline ring of TFX2 towards mildly acidic conditions and its isomerisation to the less active TFX1 form, an organism would need to produce TFX2 constantly in order to maintain its ecological niche against neighbouring α-proteobacteria. Furthermore, as the producing bacteria rarely, if ever, reach concentrations high enough to approach stationary phase in soil, to have an effect on neighbouring α-proteobacteria in soil or on plants, TFX2 needs to be made when cell concentrations are low.

## Supplementary information

Supplementary Section

## References

[1] Schwinghamer EA, Belkengren RP (1968). Inhibition of rhizobia by a strain of *Rhizobium trifolii*: some properties of the antibiotic and strain. Arch Microbiol.

[2] Triplett EW, Barta TM (1987). Trifolitoxin production and nodulation are necessary for the expression of superior nodulation competiveness by *Rhizobium leguminosarum* bv. *trifolii* strain T24 on clover. Plant Physiol.

[3] Triplett EW (1988). Isolation of genes involved in nodulation competitiveness from *Rhizobium leguminosarum* bv. *trifolii* T24. Proc Natl Acad Sci USA.

[4] Triplett EW, Breil BT, Splitter GA (1994). Expression of tfx and sensitivity to the Rhizobial peptide antibiotic trifolitoxin in a taxonomically distinct group of α-proteobacteria including the animal pathogen *Brucella abortus*. Appl Environ Microbiol.

[5] Robleto EA, Kmiecik K, Oplinger ES, Nienhis J, Triplett EW (1998). Trifolitoxin production increases nodulation competiveness of *Rhizobium elti* CE3 under agricultural conditions. Appl Environ Microbiol.

[6] Lethbridge BJ The structure of trifolitoxin: a bacteriocin from from *Rhizobium leguminosarum* b*. trifolii* strain T24. Ph.D. University of Adelaide, Australia; 1989.

[7] Roy RS, Gehring AM, Milne JC, Belshaw PJ, Walsh CT (1999). Thiazole and oxazole peptides: biosynthesis and molecular machinery. Nat Prod Rep..

[8] Arnison PG, Bibb MJ, Bierbaum G, Bowers AA, Bugni TS, Bulaj G (2013). Ribosomally synthesized and post-translationally modified peptide natural products: overview and recommendations for a universal nomenclature. Nat Prod Rep..

[9] Travin DY, Watson ZL, Metelev M, Ward FR, Osterman IA, Khven IM (2019). Structure of ribosome-bound azole-modified peptide phazolicin rationalizes its species-specific mode of bacterial translation inhibition. Nat Commun.

[10] Breil BT, Ludden PW, Triplett EW (1993). DNA sequence and mutational analysis of genes involved in the production and resistance of the antibiotic trifolitoxin. J Bacteriol.

[11] Scupham AJ, Triplett EW (2006). Determination of the amino acid residues required for the activity of the anti-rhizobial peptide antibiotic trifolitoxin. J Appl Microbiol.

[12] Hirsch PR (1979). Plasmid-determined bacteriocin production by *Rhizobium leguminosarum*. J Gen Microbiol.

[13] Bergersen FJ (1961). The growth of *Rhizobium* in synthetic medium. Aust. J Biol Sci.

[14] Scupham AJ, Dong Y, Triplett EW (2002). Role of *tfxE*, but not *tfxG*, in trifolitoxin resistance. Appl Environ Microbiol.

[15] Tate ME (1981). Determination of ionization constants by paper electrophoresis. Biochem J.

[16] Smith I Chromatographic and electrophoretic techniques. William Heinemann Medical Books, Pitman Press, Bath; 1969.

[17] Klemm P (1984). Manual Edman degradation of proteins and peptides. Methods Mol Biol.

[18] Konisberg W (1967). Subtractive Edman degradation. Meth Enzymol.

[19] Habeeb AFSA (1972). Reaction of protein sulfhydryl with Ellman’s reagent. Meth Enzymol.

[20] Hirs J (1967). Performic acid oxidation. Meth Enzymol.

[21] Frankael-Conrat H, Tsung CM (1967). Hydrazinolysis. Meth Enzymol.

[22] Smyth DG (1967). Techniques in enzymatic hydrolysis. Meth Enzymol.

[23] Greenstein JP, Winitz M Chemistry of the Amino Acids. John Wiley & Sons, New York, London; 1961.

[24] ACD/Chemsketch Freeware v2017.1.2, Advanced Chemistry Development Inc., Toronto Ontario, Canada; 2017.

[25] ProteinProspector v6.2.1, UCSF Mass Spectrometry Facility, University of California, USA; 2020.

[26] ChemBioDraw Ultra, (Version 14.0.0.117) CambridgeSoft Corp., PerkinElmer Inc., Waltham, Massachusetts, USA. (2014).

[27] Molecular Weight Calculator v6.50, Matthew Monroe, https://alchemistmatt.com; 2014.

[28] Braunschweiler L, Ernst RR (1983). Coherence transfer by isotropic mixing: Application to protein correlation spectroscopy. J Magn Reson.

[29] Bothner-By AA, Stephens RL, Lee J, Warren CD, Jeanloz RW (1984). Structure determination of a tetrasaccharide: transient nuclear Overhauser effects in the rotating frame. J Am Chem Soc.

[30] Müller L (1979). Sensitivity enhancement detection of weak nuclei using heteronuclear multiple quantum coherence. J Am Chem Soc.

[31] Bax A, Summers ML (1986). Proton and C13 assignments from sensitivity enhancement detection of heteronuclear multiple bond connectivity by 2D multiple quantum NMR. J Am Chem Soc.

[32] Rucker SP, Shaka AJ (1989). Broad-Band Homonuclear Cross Polarization. Mol Phys.

[33] FELIX 2002, Accelrys, San Diego, California, USA; 2002.

[34] Johnson BA, Blevins RA (1994). NMRView- A computer program for the visualization and analysis of NMR data. J Biomol NMR.

[35] MestReNova (Version 10.0.2-15465) Mestrelab Research S.L., Santiago de Compostela, Spain 2015.

[36] Wüthrich, K NMR of proteins and nucleic acids Wiley, New York; 1986.

[37] ChemBio3D (Version 14.0.0.117) CambridgeSoft Corporation, Perkin Elmer, Waltham, Massachusetts, USA (2014).

[38] Martin RB, Parcell A (1961). Hydrolysis of 2-substituted Δ^2^-thiazolines. J Am Chem Soc.

[39] Liu W-T Mass spectrometric and bioinformatics approaches to characterizing of cyclic non-ribosomal peptides and ribosomally encoded peptide antibiotic. MSc Thesis, UC San Diego, California, USA. 2009. (https://escholarship.org/uc/item/4jf327w7)

[40] Przychodzeń W (2006). Lawesson’s reagent for direct thionation of hydroxamic acids: substituent effects on LR reactivity. Heteroat Chem.

[41] Brown DA, Coogan RA, Fitzpatrick NJ, Glass WK, Abukshima DE, Shiels L (1996). Conformational behaviour of hydroxamic acids: ab initio and structural studies. J Chem Soc, Perkin Trans.

[42] Bunting JW (1980). Heterocyclic pseudobases. Adv Heterocycl Chem.

[43] Perrin DD, Dempsey B, Serjeant EP 1981. pKa prediction for organic acids and bases. Chapman and Hall, London, UK and New York, USA; 1981.

[44] Schmir GL (1965). The Effect of Structural Variation on the Hydrolysis of Δ2-Thiazolines. J Am Chem Soc.

[45] Scott AI Interpretation of the ultraviolet spectra on natural products, Pergamon Press, Oxford, London, Edinburgh, New York, Paris, Frankfurt; 1964.

[46] Cody CW, Prasher DC, Westler WM, Prendergast FG, Ward WW (1993). Chemical structure of the hexapeptide chromophore of the *Aequorea* green-fluorescent protein. Biochem.

[47] Houwaart S, Youssar L, Hüttel W (2014). Pneumocandin biosynthesis: involvement of a *trans*-selective proline hydroxylase. ChemBioChem.

[48] Takemoto T, Nakajima T (1964). Structure of tricholomic acid. Yakugaku Zasshi.

[49] Takemoto T, Nakajima T, Sakuma R (1964). Isolation of a flycidal constituent “ibotenic acid” from *Amanita muscaria* and *A. pantherina*. Yakugaku Zasshi.

[50] Obermaier S, Müller M (2020). Ibotenic acid biosynthesis in the fly agaric is initiated by glutamate hydroxylation. Angew Chem Int.

[51] Lechan RM, Wu P, Jackson IM, Wolf H, Cooperman S, Mandel G (1986). Thyrotropin-releasing hormone precursor: characterization in rat brain. Science.

[52] Richter K, Kawashiima E, Egger R, Kreil G (1984). Biosynthesis of thyrotropin releasing hormone in the skin of *Xenopus laevis*: partial sequence of the precursor deduced from cloned cDNA. EMBO J.

[53] Seeburg PH, Adelman JP (1984). Characterization of a cDNA precursor of human luteinizing hormone-releasing hormone. Nature.

[54] Cubitt AB, Heim R, Adams SR, Boyd AE, Gross LA, Tsien RY (1995). Understanding, improving and using green fluorescent proteins. Trends Biochem Sci.

[55] Ishihara H, Sasaki T, Shimura K (1968). Biosynthesis of bacitracin: II. Incorporation of 14C-labeled amino acids into bacitracin by a cell-free preparation from *Bacillus licheniformis*. Biochim Biophys Acta, Nucleic Acids Protein Synth.

[56] Anderson B, Hodgkin D, Viswamitra M (1970). The structure of thiostrepton. Nature.

[57] Kelly WL, Pan L, Li C (2009). Thiostrepton biosynthesis: prototype for a new family of bacteriocins. J Am Chem Soc.

