# Supplementary material for: Post translational modifications of Trifolitoxin: a blue fluorescent peptide antibiotic
Source: J Antibiot (Tokyo). 2022 Jan 12;75(3):125–35. doi: 10.1038/s41429-021-00497-0 (PMC8816728; doi:10.1038/s41429-021-00497-0)
Supplement: Supplementary file 1 — Supplementary Section [file 41429_2021_497_MOESM1_ESM.docx]

**Supplementary Section**

Index S1

Peptide analysis S2

UV-Vis spectra S3

HPLC chromatogram S6

Mass spectroscopy (MS) S7

FAB-MS

High Resolution MS

(+)-ESI-MS

(-)-ESI-MS

Infrared spectrum S27

NMR S28

Proton ^1^H

Cosy

HSQC

HMBC

HMBC

Tocsy

Roesy

Biosynthetic pathway of TFX S40

Molecular models S43

TFX2 (trans-TFX)

TFX1 (cis-TFX)

References S45

**Peptide Analysis**

Figure S1. Chemical analyses of TFX peptide

**UV-Visible spectrum**

Figure S2. UV-vis spectra of TFX1 (dashed line; HPLC retention time 11.2min) and TFX2 (solid line; HPLC retention time (11.8min)

Figure S3. TFX2 (A) UV absorbing (239 and 302 nm at pH 7) chromophore and (B) blue fluorescent chromophore (excitation wavelength 254nm)


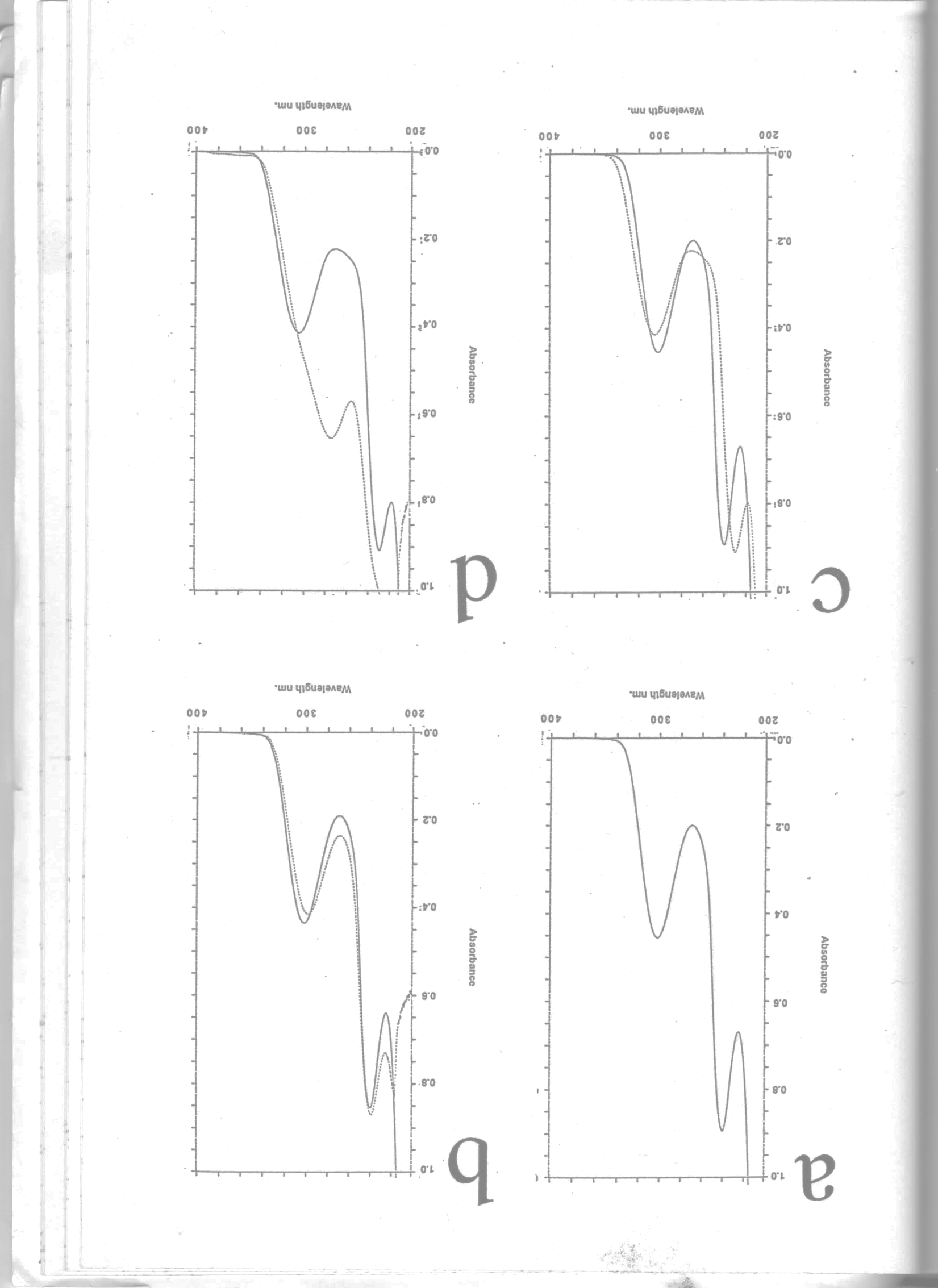


Figure S4. Absorbance spectra of TFX (a) pH 7.0 (solid) compared with (b) pH 13.0 (dashed), (c) pH 1.0 (dashed) and (d) pH -1.0 (dashed)


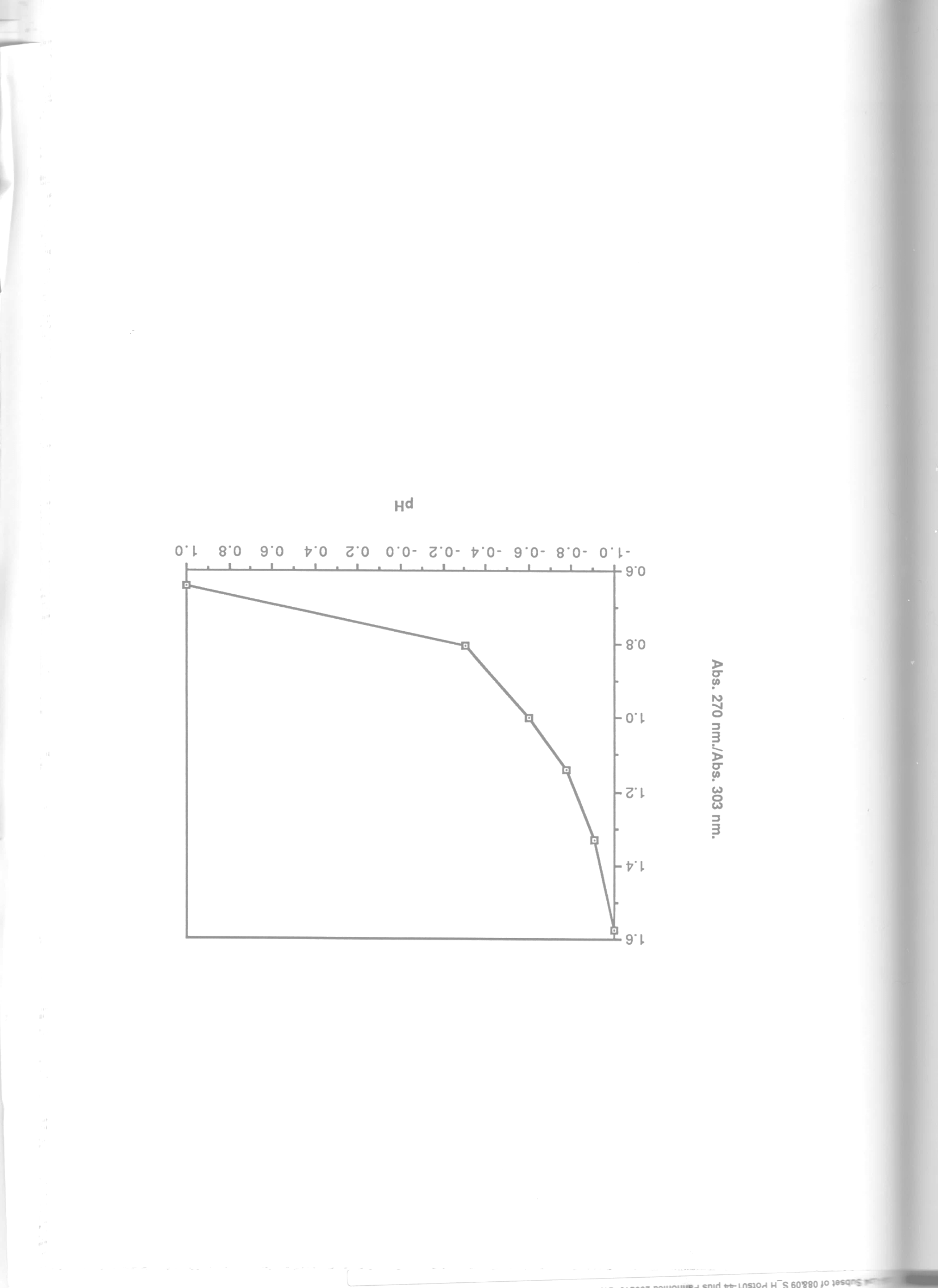


Figure S5. Ratio of 270nm/304nm UV absorbance as a function of pH


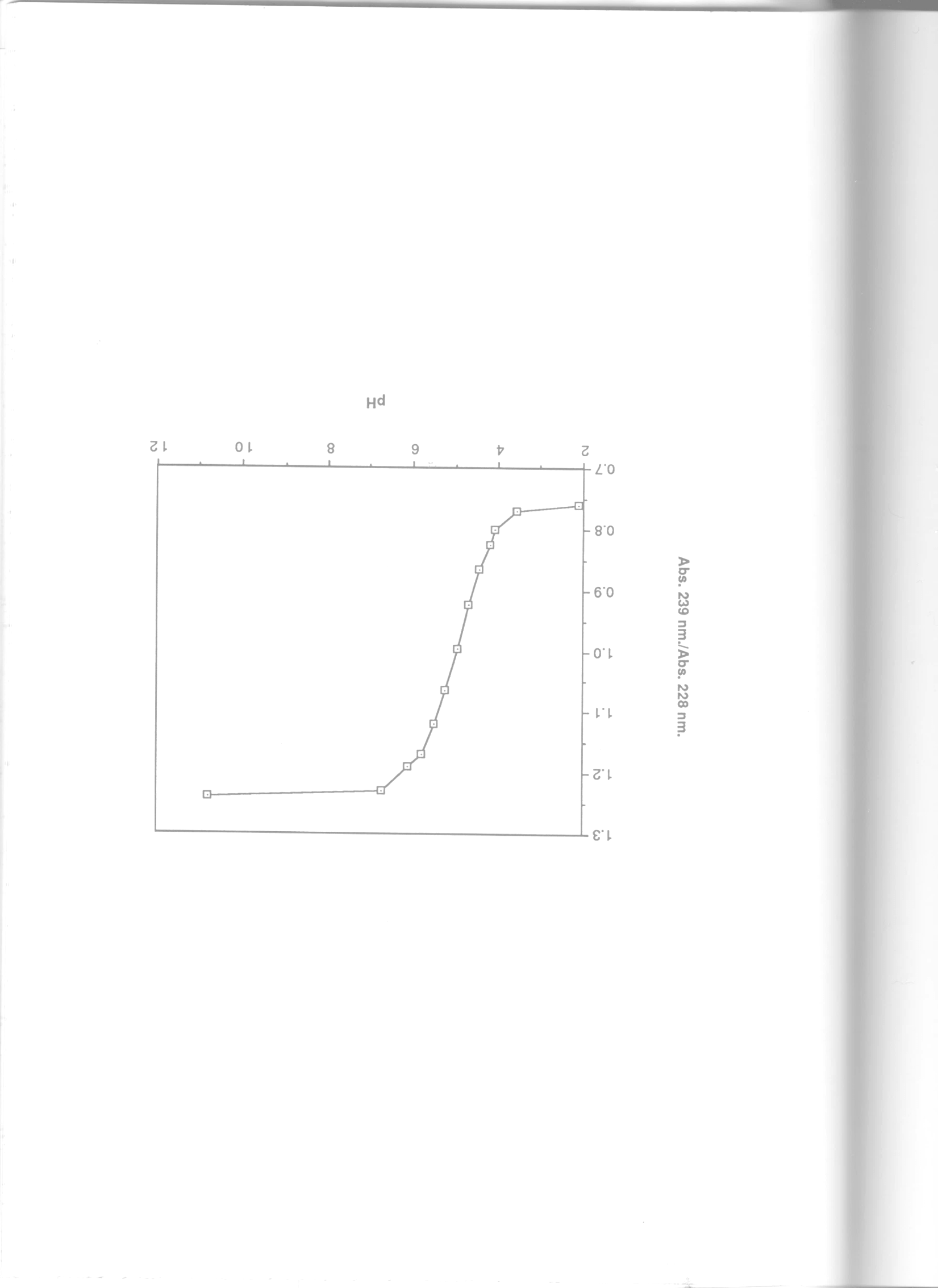


Figure S6. Ratio of the absorbance at 239nm/228nm compared with pH

**HPLC chromatogram**

Figure S7. Chromatogram showing separation of TFX1 and TFX2 by Hewlett-Packard 1090 LC using a Vydac protein/peptide C18 column (5um, 250x2.1mm) and eluents (A) 10mM triethylammonium acetate pH 6.5 and (B) 10mM triethylammonium acetate in 40% acetonitrile. Gradient was 15-25% B over 20min, and flow rate of 0.2mL/min at 21^o^C. Detection at 304nm.

**Mass Spectrometry**

Figure S8. Fast atom bombardment mass spectrometry (FAB-MS) ionisation pattern of TFX. Data obtained on a VG-ZABN-T four sector Mass Spectrometer (VG Analytical Ltd, Manchester, UK) using an array detector. Fragments were assigned according to Roepstorff and Fohlman (1984). From these data it could be established that the N-terminus reads Asp-Ile-Gly-Gly-Ser. TFX-1 and TFX-2 are isomers, do not differ near the N-terminus and both TFX1 and TFX2 have α-Asp as the N-terminal amino acid. Many of the ions could not be assigned (Table S1).

It should be noted that all mass spectra are of the anhydro forms of TFX1 and TFX2.

Table S1. TFX (m/z 1038 (M+H^+^)) MS/MS daughter ions obtained by FAB-MS and some suggested assignments.

| Ion MS (d+H) ^+^ | Relative Intensity | Assignment |
| --- | --- | --- |
| 86 | 0.83 |  |
| 88 | 0.17 | a1 |
| 115 | 0.04 |  |
| 201 | 0.94 | a2 |
| 220 | 0.71 |  |
| 229 | 0.06 | b2 |
| 245 | 0.25 | c2 |
| 262 | 0.33 |  |
| 286 | 0.15 | b3 |
| 316 | 0.18 | a4 |
| 341 | 0.10 |  |
| 343 | 0.07 | b4 |
| 359 | 0.04 | c4 |
| 415 | bp |  |
| 440 | 0.13 |  |
| 457 | 0.17 |  |
| 475 | 0.07 |  |
| 494 | 0.05 |  |
| 544 | 0.05 |  |
| 574 | 0.09 |  |
| 592 | 0.22 | z6 |
| 609 | 0.14 | y6 |
| 645 | 0.04 |  |
| 679 | 0.09 | z7 |
| 689 | 0.34 |  |
| 708 | 0.30 |  |
| 725 | 0.23 | c7 |
| 764 | 0.06 |  |
| 821 | 0.52 | a9 |
| 838 | 0.13 | x10 |
| 867 | 0.11 | c9 |
| 902 | 0.33 |  |
| 922 | 0.37 | y11 |
| 937 | 0.28 |  |
| 948 | 0.11 | b10 |

TFX_MRM-MSMS-02_GC5_01_6379.d: EIC 519.72000±0.05 +All MS

0

1000

2000

3000

4000

Intens.

10

20

30

40

50

60

70

Time [min]

Figure S9. Liquid chromatographic separation of TFX1 and TFX2 for high resolution mass spectrometry (HRMS) showing the extracted mass chromatogram of m/z 519.72, the [M+2H]^2+^ ion of TFX isomers. Separation was performed with a Thermo Scientific Ultimate 3000 series HPLC (Thermo Scientific, Waltham, Massachusetts, U.S.) consisting of degasser, binary pump, column compartment and autosampler controlled by the Dionex Chromeleon Xpress software (Thermo Scientific). Chromatographic separation was achieved on a ThermoScientific Acclaim PepMap RSLC (300 µm x 15 cm, 2 µm, 100 Å) with a flow rate of 5 microliters/min at 40C column temperature. Mobile phases A and B were water and methanol, respectively, each with 10 mM ammonium formate and 0.1% formic acid. The following gradient was used: 2%B (0-2 min) increased to 10%B (21 min) increased to 95%B (40 min) and held at 95%B for 10 min

Figure S10. High Resolution mass spectrum of TFX1 showing the m/z 519.72309 [M+2H]^2+^ ion being the dominant ion. TFX+H_2_O m/z 528.72903 [M+2H]^2+^

(Relative intensity 0.14) was also observed. It should be noted that TFX can hydrate across the arginine amide/arginine α-carbon double bond to form a hydroxamic acid and at the thiazoline ring to form the pseudobase (pK_H_ 2.61 ± 0.05). The ion m/z 695.8 is not related to TFX1. High resolution mass spectra were acquired on a Bruker Impact II UHR-QqTOF-MS (Ultra-High-Resolution Qq-Time-of-Flight; Bruker Daltonik GmbH, Bremen, Germany ) controlled by the Hystar Software 3.2 SR4 (version 3.2 Build 49.9; Bruker Daltonik GmbH) and operated in the positive ESI mode with 4.5 kV ionization , 4.0 L/min nitrogen drying gas at 200C and a nebulizer pressure of 0.3 bar. Mass calibration was achieved by infusion with syringe pump (Model 601553, KD Scientific, Holliston, MA) of a sodium formate solution in the first 0.9 min of each analysis with subsequent recalibration of all data acquired after this time. Data-dependent MS/MS spectra were acquired at 25 eV with 1 mtorr nitrogen collision gas with 5-u isolation of the precursor ion.

Figure S11 High Resolution mass spectrum of TFX2 showing the m/z 519.72309 [M+2H]^2+^ ion being the dominant ion. TFX+H_2_0 m/z 528.72885 [M+2H]^2+^

(Relative intensity 0.06) was observed. The m/z 695.8 is not related to TFX2. High resolution mass spectra were acquired on a Bruker Impact II UHR-QqTOF-MS (Ultra-High-Resolution Qq-Time-of-Flight; Bruker Daltonik GmbH) controlled by the Hystar Software 3.2 SR4 (version 3.2 Build 49.9; Bruker Daltonik GmbH) and operated in the positive ESI mode with 4.5 kV ionization , 4.0 L/min nitrogen drying gas at 200C and a nebulizer pressure of 0.3 bar. Mass calibration was achieved by infusion with syringe pump (Model 601553, KD Scientific, Holliston, MA) of a sodium formate solution in the first 0.9 min of each analysis with subsequent recalibration of all data acquired after this time. Data-dependent MS/MS spectra were acquired at 25 eV with 1 mtorr nitrogen collision gas with 5-u isolation of the precursor ion.

Table S2. Estimation of the mass of TFX1 and TFX2 by High resolution mass spectrometry. A theoretical mass of m/z 519.72471, [M+2H]^2+^, gives a molecular formula, C_41_H_65_N_15_O_15_S^2+^

| **TFX-1** |  |  |  | **TFX-2** |  |  |
| --- | --- | --- | --- | --- | --- | --- |
| **RT** | **m/z** | **error (ppm)** |  | **RT** | **m/z** | **error**  **(ppm)** |
| 25.82 | 519.72368 | -1.98 |  | 26.08 | 519.72435 | -0.69 |
| 25.87 | 519.72472 | 0.02 |  | 26.13 | 519.72381 | -1.73 |
| 25.93 | 519.72309 | -3.12 |  | 26.19 | 519.72426 | -0.87 |
| 25.98 | 519.72439 | -0.62 |  | 26.24 | 519.72363 | -2.08 |
| 26.03 | 519.72424 | -0.90 |  | 26.29 | 519.72400 | -1.37 |
|  | 519.72000 | -9.06 |  | 26.34 | 519.72334 | -2.64 |
|  | 519.72000 | -9.06 |  | 26.4 | 519.72327 | -2.77 |
|  |  |  |  | 26.45 | 519.72408 | -1.21 |
|  |  |  |  | 26.5 | 519.72397 | -1.42 |
|  |  |  |  | 26.55 | 519.72544 | 1.40 |
|  |  |  |  | 26.6 | 519.72236 | -4.52 |
|  |  |  |  | 26.66 | 519.72492 | 0.40 |
|  |  |  |  |  |  |  |
| Average | 519.72287 | -3.53 |  |  | 519.72395 | -1.46 |
| STd Dev | 0.00203 | 3.91 |  |  | 0.00079 | 1.53 |
| %RSD | 0.00039 |  |  |  | 0.00015 |  |
| count | 7 | 7 |  |  | 12 | 12 |
|  |  |  |  |  |  |  |
| Theory | 519.72471 |  |  | Theory | 519.72471 |  |
| Average | 519.72287 |  |  | Average | 519.72395 |  |
| ppm | -3.54 |  |  | ppm | -1.46 |  |

Figure S12. (+)ESI-MS/MS high resolution product spectrum of m/z 519.7 [M+2H]^2+^ ion of TFX1

Figure S13. (+)ESI-MS/MS high resolution product spectrum of the m/z 519.7 [M+2H]^2+^ ion of TFX2.

Figure S14. Structure of TFX with the conventional cleavages for the formation of b and y product ions with the expected m/z value. When 2 ions are given, the upper is the z = +1 ion and the lower is the z = +2 ion. The structure is shown as a neutral. Internal cleavages (rearrangements via loss of amino acids residues from either end) also were noted. The numbering was maintained according to the unmodified amino acid sequence. In later Tables, ion identification utilized the NH2-D-I-G-G-S-u-V-A-COOH shorthand where the “Rx6, Qy7, G8-G9” have been replaced with “u”.

Table S3. Product ions produced by the (+)-ESI-MS/MS of m/z 519.7 [M+2H]^2+^ ion of the TFX isomers.

|  |  |  |  | **TFX-1** |  |  |  |  |  | **TFX-2** |  |  |  |
| --- | --- | --- | --- | --- | --- | --- | --- | --- | --- | --- | --- | --- | --- |
| **Ion, m/z** | **z** |  | **Avg, m/z** | **SD, m/z** | **SD, ppm** | **Count** | **%RA** |  | **Avg, m/z** | **SD, m/z** | **SD, ppm** | **Count** | **%RA** |
| 949.4 | 1 |  | 949.39057 | 0.00325 | 3.42 | 3 | 4.7 |  | 949.39432 | 0.00179 | 1.88 | 3 | 2.3 |
| 931.4 | 1 |  | 931.38125 | 0.00315 | 3.38 | 2 | 3.7 |  | 931.38275 | 0.00158 | 1.70 | 4 | 3.3 |
| 810.3 | 1 |  | 810.33019 | 0.00116 | 1.43 | 6 | 20.3 |  | 810.33026 | 0.00168 | 2.08 | 9 | 14.1 |
| 792.3 | 1 |  | 792.31803 | 0.00136 | 1.72 | 6 | 29.0 |  | 792.31793 | 0.00177 | 2.23 | 11 | 26.8 |
| 719.3 | 1 |  | 719.32002 | 0.00240 | 3.34 | 4 | 5.1 |  | 719.32185 | 0.00188 | 2.61 | 6 | 6.8 |
| 693.3 | 1 |  | 693.28610 | 0.00205 | 2.95 | 5 | 10.3 |  | 693.28817 | 0.00221 | 3.19 | 6 | 7.0 |
| 675.3 | 1 |  | 675.27633 | 0.00083 | 1.23 | 6 | 32.7 |  | 675.27598 | 0.00127 | 1.88 | 10 | 29.6 |
| 658.2 | 1 |  | 658.24882 | 0.00299 | 4.54 | 2 | 3.8 |  | 658.25092 | 0.00193 | 2.93 | 3 | 2.3 |
| 639.2 | 1 |  | 639.24061 | 0.00159 | 2.48 | 4 | 6.3 |  | 639.24080 | 0.00226 | 3.53 | 6 | 5.8 |
| 621.2 | 1 |  | 621.23014 | 0.00230 | 3.71 | 5 | 8.6 |  | 621.22919 | 0.00132 | 2.12 | 8 | 8.7 |
| 510.7 | 2 |  | 510.71834 | 0.00301 | 5.90 | 4 | 6.4 |  | 510.71727 | 0.00067 | 1.31 | 4 | 3.6 |
| 466.2 | 2 |  | 466.19554 | 0.00098 | 2.09 | 2 | 6.2 |  | 466.19563 | 0.00272 | 5.84 | 4 | 3.2 |
| 461.2 | 2 |  | 461.20135 | 0.00062 | 1.34 | 4 | 10.9 |  | 461.20231 | 0.00246 | 5.33 | 8 | 7.5 |
| **452.2** | **2** |  | **452.19699** | **0.00033** | **0.73** | **6** | **100.0** |  | **452.19662** | **0.00041** | **0.90** | **11** | **100.0** |
| 443.2 | 2 |  | 443.19076 | 0.00136 | 3.06 | 5 | 11.2 |  | 443.19087 | 0.00173 | 3.90 | 7 | 10.5 |
| 435.2 | 2 |  | 435.20177 | 0.00116 | 2.67 | 4 | 8.3 |  | 435.20237 | 0.00185 | 4.24 | 6 | 3.9 |
| 394.7 | 2 |  | 394.68318 | 0.00176 | 4.46 | 5 | 6.4 |  | 394.68342 | 0.00090 | 2.28 | 6 | 6.3 |
| 360.2 | 2 |  | 360.16340 | 0.00062 | 1.73 | 3 | 5.3 |  | 360.16228 | 0.00184 | 5.12 | 4 | 3.0 |
| 338.1 | 2 |  | 338.14147 | 0.00076 | 2.24 | 5 | 15.3 |  | 338.14184 | 0.00144 | 4.26 | 8 | 13.8 |

Table S4. Assignment of structures, elemental compositions to the various product ions of the (+)-ESI MS/MS of m/z 519.7 [M+2H]^2+^ ion of TFX2. The blue shaded product ions were present in the spectra but were minor ions.

| **Ion type** | **Precursor ion, [M+2H]2+** | **Elemental composition** | **Theoretical monoisotopic m/z** | **TFX-2 Product ions** | **Delta, m/z** | **Delta, ppm** |
| --- | --- | --- | --- | --- | --- | --- |
| b10 | NH_2_-D-I-G-G-S-u-V-CO^+^ | [C_38_H_57_N_14_O_13_S]^+^ | 949.39447 | 949.39432 | -0.00015 | -0.16 |
| b10-H_2_O | [NH_2_-D-I-G-G-S-u-V-CO]^+^-H_2_O | [C_38_H_55_N_14_O_12_S]^+^ | 931.38391 | 931.38275 | -0.00116 | -1.24 |
| y9 | [NH_2_-G-G-S-u-V-A-COOH]^+^ | [C_31_H_48_N_13_O_11_S]^+^ | 810.33115 | 810.33026 | -0.00089 | -1.10 |
| y9-H_2_O | [NH_2_-G-G-S-u-V-A-COOH]^+^-H_2_O | [C_31_H_46_N_13_O_10_S]^+^ | 792.32058 | 792.31793 | -0.00264 | -3.34 |
| y8 | [NH_2_-G-S-u-V-A-COOH]^+^ | [C_29_H_45_N_12_O_10_S]^+^ | 753.30968 | 753.30911 | -0.00057 | -0.76 |
| y8-H_2_O | [NH_2_-G-S-u-V-A-COOH]^+^-H_2_O | [C_29_H_43_N_12_O_9_S]^+^ | 735.29912 | 735.29771 | -0.00141 | -1.92 |
|  |  |  |  | 719.32185 |  |  |
| Int clv | [NH_2_-G-G-S-u-V-CO]^+^-CO | [C_27_H_41_N_12_O_8_S]^+^ | 693.28855 | 693.28817 | -0.00038 | -0.55 |
| Int clv | [NH_2_-G-G-S-u-V-CO]^+^-CO-H_2_O | [C_27_H_39_N_12_O_7_S]^+^ | 675.27798 | 675.27598 | -0.00200 | -2.96 |
| Int clv | [NH_2_-G-G-S-u-V-CO]^+^-CO-H_2_O-NH_3_ | [C_27_H_36_N_11_O_7_S]^+^ | 658.25144 | 658.25092 | -0.00052 | -0.78 |
|  |  |  |  | 639.24080 |  |  |
|  |  |  |  | 621.22919 |  |  |
| y11-H_2_O | [M+2H-H2O]^2+^ | [C_41_H_63_N_15_O_14_S]^2+^ | 510.71943 | 510.71727 | -0.00216 | -4.23 |
| b10-H_2_O | [NH_2_-D-I-G-G-S-u-V-CO]^2+^-H_2_O | [C_38_H_56_N_14_O_12_S]^2+^ | 466.19559 | 466.19563 | 0.00004 | 0.09 |
| a10 | [NH_2_-D-I-G-G-S-u-V-CO]^2+^ -CO | [C_37_H_58_N_14_O_12_S]^2+^ | 461.20342 | 461.20231 | -0.00111 | -2.40 |
| a10-H_2_O | [NH_2_-D-I-G-G-S-u-V-CO]^2+^-CO-H_2_O | [C_37_H_56_N_14_O_11_S]^2+^ | 452.19813 | 452.19662 | -0.00151 | -3.34 |
| a10-2H_2_O | [NH_2_-D-I-G-G-S-u-V-CO]^2+^ -CO-2H_2_O | [C_37_H_54_N_14_O_10_S]^2+^ | 443.19285 | 443.19087 | -0.00198 | -4.46 |
|  |  |  |  | 435.20237 |  |  |
|  |  |  |  | 394.68342 |  |  |
|  | z=+2 ion of m/z 719 |  |  | 360.16228 |  |  |
|  |  |  |  | 338.14184 |  |  |

Figure S15. Summary of the low resolution MS/MS of TFX2 b and y ions where the red ions were not observed (JVJ 2003, 2008). Some examples are shown below (pp S17-S23)

Figure S16. Comparison of the HPLC/UV (302 nm) chromatograms of TFX-1 (middle), and TFX-2(bottom) and a mixture of the two (top). Separation achieved by Agilent (Palo Alto, CA, USA) 1100 series binary pump using an Atlantis dC18 column (2.1x150mm, 3μm, Waters Corporation, Milford, Massachusetts). Mobile phase was (A) 0.2% HCOOH in water (B) 0.2% HCOOH in methanol with a gradient of (A) starting from 90% to 75% at 10 min then 65% at 40 min run at 25^o^C. A 25μL injection was used.

Figure S17. Comparison of (+)-ESI-MS/MS of m/z 519.7 [M+2H]^2+^ ions from TFX1 (bottom) and TFX2 (top). Mass spectrum obtained using a ThermoFinnigan (San Jose, CA. USA) LCQ in electrospray ionisation (ESI) mode.)

Table S5. Comparison of TFX1 and TFX2 isomer daughter ions their suggested assignments obtained by (+)-ESI-MS/MS of 1038 [M+H]^+^. Data from figures S12 & S13

|  | TFX1 | Intensity | TFX2 | Intensity | d^+^ | d^++^ |  |
| --- | --- | --- | --- | --- | --- | --- | --- |
|  | 931.2 | 0.03 | 931.2 | 0.04 | b10-H_2_O |  | |
|  | 810.2 | 0.08 | 810.2 | 0.07 | y9 |  | |
|  | 792.1 | 0.05 | 792.2 | 0.05 | y9-H_2_O |  | |
|  | 690.3 | 0.03 | 690.1 | 0.01 | b10-x9 |  | |
|  | 675.1 | 0.03 | 675.2 | 0.03 | z8-CO_2_-H_2_O |  | |
|  | 510.6 | 1.00 | 510.6 | 1.00 |  | TFX-H_2_O | |
|  | 501.7 | 0.18 | 501.7 | 0.13 |  | TFX-2H_2_O | |
|  | 466.1 | 0.73 | 466.1 | 0.76 |  | b10-H_2_O | |
|  | 452.2 | 0.50 | 452.2 | 0.75 |  | a10-H_2_O | |
|  | 430.0 | 0.01 | 430.1 | 0.01 | b5 |  | |
|  | 405.5 | 0.04 | 405.6 | 0.04 |  | y9 | |
|  | 396.6 | 0.02 | 396.6 | 0.02 |  | y9-H_2_O | |
|  | 331.0 | 0.01 | 330.9 | 0.01 | y4-H_2_O |  | |
|  | 228.8 | 0.03 | 228.9 | 0.03 | b2 |  | |
|  | 200.9 | 0.02 | 201.0 | 0.03 | a2 |  | |

Figure S18 Diagram indicating the position of the major fragment ions obtained by (+)-ESI-MS/MS of the m/z 519.7 [M+2H]^2+^ ions (See table above)

Figure S19. Chromatograms of the separation of TFX-1 and TFX-2 by Thermo Scientific Ultimate 3000 HPLC with dual pumps, column heater and auto-sampler using a Thermo-Scientific HyperSil Gold aQ (2.1 x 150 mm; 3um; + guard column) column at 35^o^C. Mobile phases were (A) 10mM ammonium acetate and 0.1% formic acid in water and (B) 10mM ammonium acetate and 0.1% formic in water:methanol (1:9) Gradient (@ 0.2 mL/min) was 5%B at 0 min increased to 30%B at 37.5 min and then to 95%B at 60 min and then held at 95%B for 10 min).

Figure S20 TFX1: (+)ESI-MS/MS of m/z 519 [M+2H]^2+^ ions obtained using a ThermoScientific LTQ XL linear quadrupole ion trap mass spectrometer with electrospray ionization (ESI) operating with XCALIBUR 2.2 SP1.48.

Figure S21 TFX2: (+)ESI-MS/MS of m/z 519 [M+2H]^2+^ ions obtained using a ThermoScientific LTQ XL linear quadrupole ion trap mass spectrometer with electrospray ionization (ESI) operating with XCALIBUR 2.2 SP1.48

Table S6. Comparison of (+)-ESI-MS/MS product ions of m/z 519.7 [M+2H]^2+^ ions from TFX1 and TFX2 isomers and their suggested assignments. Data from figures S15-S17

|  | TFX1 | Intensity | TFX2 | Intensity | d^+^ | d^++^ |  |
| --- | --- | --- | --- | --- | --- | --- | --- |
|  | 931.4 | 0.03 | 931.2 | 0.04 | b10-H_2_O |  | |
|  | 810.4 | 0.08 | 810.2 | 0.07 | y9 |  | |
|  | 792.4 | 0.05 | 792.2 | 0.05 | y9-H_2_O |  | |
|  | 753.4 | 0.00 | 753.4 | 0.00 | y8 |  | |
|  | 719.6 | 0.01 | 719.6 | 0.06 | x8-CO_2_-H_2_O |  | |
|  | 690.3 | 0.04 | 690.1 | 0.01 | b10-x9 |  | |
|  | 675.1 | 0.03 | 675.2 | 0.03 | z8-CO_2_-H_2_O |  | |
|  | 510.8 | 0.26 | 510.8 | 0.16 |  | TFX-H_2_O | |
|  | 501.9 | 0.18 | 501.9 | 0.22 |  | TFX-2H_2_O | |
|  | 475.2 | 0.15 | 475.2 | 0.16 |  | b10 | |
|  | 466.3 | 1.00 | 466.3 | 1.00 |  | b10-H_2_O | |
|  | 452.3 | 0.44 | 452.4 | 0.50 |  | a10-H_2_O | |
|  | 405.8 | 0.06 | 405.9 | 0.04 |  | y9 | |

Figure S22 Diagram indicating the position of the major fragment ions obtained by (+)-ESI-MS/MS of m/z 519.7 [M+2H]^2+^ (See table above)

Figure S23 TFX high resolution mass spectrum obtained by reverse phase LC/MS. Spectra were obtained using a Brucker microQTOF-Q mass spectrometer with (-)-ESI (electrospray ionisation) Expected 1036.4276 m/z [M-H]^-^ for C_41_H_62_N_15_O_14_S^-^, obtained 1036.4240 m/z. Data obtained by Mark Solomon, Australian Wine Research Institute-Metabolomics, Urrbrae, SA Australia

**Infra-red spectrum**

Figure S24. Infrared (IR) spectrum of TFX obtained on a Perkin Elmer Spectrum 1 spectrometer by diffuse reflectance. IR spectrum is consistent with a peptide.

**Nuclear Magnetic Resonance (NMR)**

Figure S25 TFX1 ^1^H NMR in D_2_O at 600 MHz

Figure S26 TFX2 ^1^H NMR in D_2_O at 600 MHz

**_TFX1_**

**_TFX2_**


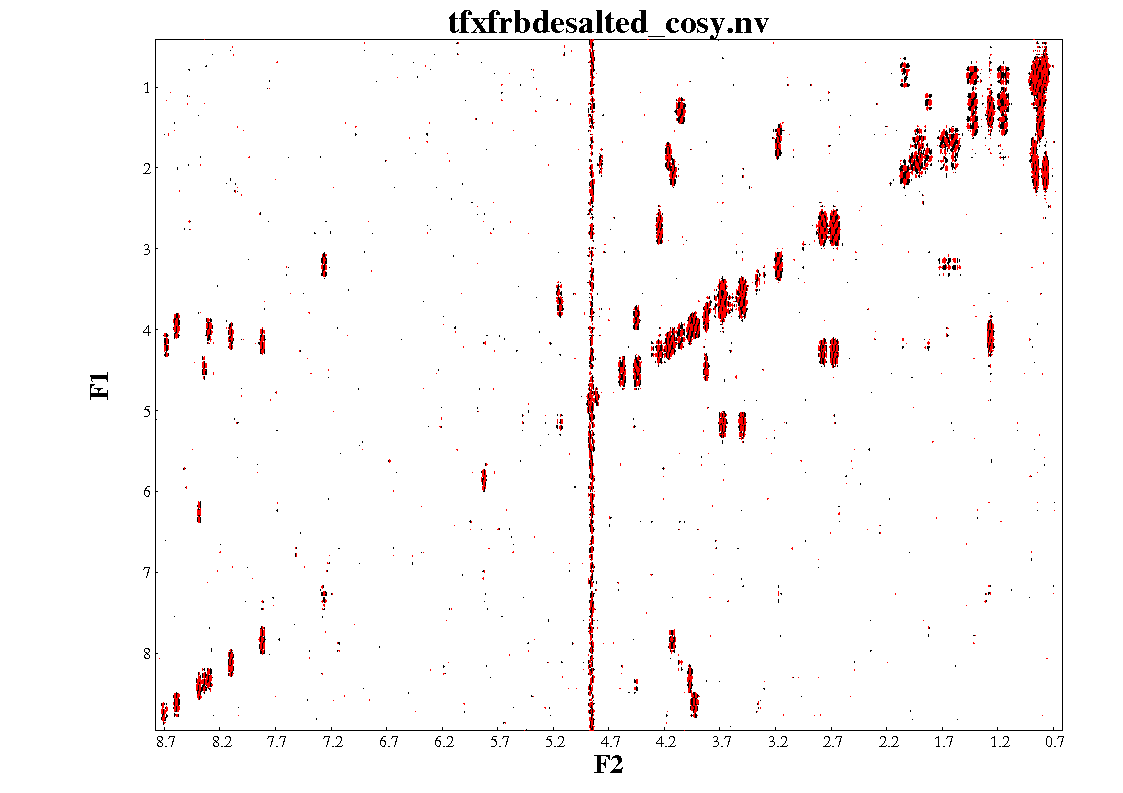


S5

G3

I2

G4

A11

V10

R6 guandinium

H 5.82

H 5.14

F2 (^1^H ppm)

F1 (^1^H ppm)

Figure S27. TFX2 COSY (^1^H-^1^H Correlated Spectroscopy) spectrum

Figure S28. TFX1 HSQC (^1^H-^13^C Heteronuclear Single Quantum Coherence) spectrum

Figure S29. TFX2 HSQC (^1^H-^13^C Heteronuclear Single Quantum Coherence) spectrum

Figure S30. TFX1 ^1^H-^13^C HSQC spectrum showing assignments

Figure S31. TFX1 HMBC (^1^H-^13^C Heteronuclear Multiple Bond Correlation) spectrum

Figure S32. TFX2 HMBC (^1^H-^13^C Heteronuclear Multiple Bond Correlation) spectrum

Figure S33. TFX2 TOCSY (^1^H-^1^H Total Correlation Spectroscopy) spectrum showing correlations

Figure S34. TFX1 TOCSY (^1^H-^1^H Total Correlation Spectroscopy) spectrum showing correlations

Figure S35. TOCSY NH region of TFX1 & TFX2 hydroxamic acid cis-isomers. Arginine proton NMR shift values observed for TFX1 and TFX2 (8.91, 8.95ppm) compared with the usual values (8.52ppm, 8.53ppm) was consistent with a hydroxamic acid cis-isomer. TFX1 has only protons associated with the open thiazoline ring (C9*, V10*, A11*) while TFX2 has proton values for both open (C9*, V10*, A11*) and closed (V10, A11) thiazoline ring forms. A downfield shift of the isoleucine amide proton from I2 (TFX1, 8.69ppm; TFX2, 8.70ppm) to I2# (TFX1, 8.78ppm; TFX2, 8.79ppm) indicates hydrogen bonding of the amide carbonyl to the aspartic acid carboxyl proton. The presence of two isoleucine isomers suggests the aspartate carboxyl (pKa ≈4) was partially protonated.

Figure S36. TFX1 Roesy (^1^H-^1^H Rotating Frame Nuclear Overhauser Effect Spectroscopy) spectrum

Figure S37. TFX2 ROSEY (^1^H-^1^H Rotating frame nuclear Overhauser effect spectroscopy) spectrum

Figure S38. TFX-2 ROESY correlation (a) between the chromophore proton (5.8ppm) and a proton at 4.51ppm is consistent with a single serine α-proton. (b) and (c) indicate the serine α-proton (4.51ppm) correlates with one NH proton (8.41ppm) and two protons at 3.86ppm (serine β-protons), respectively. Isomerisation occurred during data accumulation and so the serine NH and α-protons are split. Noe correlation between the serine α-proton and the chromophore proton indicated on the structure.

Figure S39. Biosynthetic pathway of TFX chromophore

**Molecular Models**

Figure S40. MM2 model of TFX2 hydrate showing the relative positions of the chromophore proton and the serine α-proton

Figure S41. MM2 model of TFX1 hydrate showing the relative positions of the chromophore proton and the serine α-proton

**References**

Roepstorff P, Fohlman J. Proposal for a common nomenclature for sequence ions in mass spectra of peptides. J. Biomed. Mass Spectrom. 1984; 11: 601.
